# A randomized controlled trial in healthy participants to compare the insulinogenic effects of whey protein and pea protein co-ingested with glucose

**DOI:** 10.1371/journal.pone.0340386

**Published:** 2026-01-30

**Authors:** Pariyarath Sangeetha Thondre, Elysia Young, Sam Pledger, Sarah Kefyalew, Isabel Hatami, Caroline Perreau, Laetitia Guérin Deremaux, Catherine Lefranc-Millot, Jonathan Tammam

**Affiliations:** 1 Oxford Brookes Centre for Nutrition and Health, Faculty of Health, Science and Technology, Oxford Brookes University, Oxford,; 2 Roquette, Life Sciences R&D., Lestrem, France; Periyar University, INDIA

## Abstract

Increasing protein content of foods is effective in reducing postprandial hyperglycaemia, but animal protein may exacerbate insulin sensitivity. This single-blind, randomised, crossover study compared the effects of co-ingesting glucose with 10 or 20 g whey protein and glucose with 10 or 20 g pea protein, with a reference product (glucose) on glycaemic and insulinaemic responses in 30 healthy individuals. Blood glucose and plasma insulin were measured at baseline, 15, 30, 45, 60, 90, 120, 150 and 180 minutes after product consumption. The trial was registered with Clinical Trials.gov (NCT04871971). Glucose incremental area under the curve (mmol/l*min) at 180 minutes was significantly reduced (p < 0.001) for glucose with 20 g pea protein (89.8 ± 51.6) and glucose with 20g whey protein (98.5 ± 58.0) compared to glucose (143.2 ± 74.0). Insulin incremental area under the curve at 180 minutes (µU/ml*min) for glucose with 20 g pea protein (4304.56 ± 1896.07) was significantly lower (p < 0.001) than glucose with 20g whey protein (6311.81 ± 3489.12). This study has shown a superior effect of pea protein over whey protein in reducing glycaemic response, without any excessive increase in insulinaemic response.

## 1. Introduction

Type 2 Diabetes (T2D) is a non –communicable disease that continues to affect millions of individuals worldwide with pre-diabetes being the intermediate stage between normoglycaemia and T2D [1]. T2D prevalence affected 10.5% of the global adult population in 2021 with predictions to increase to 12.2% by 2045 [2]. Individuals with T2D are at increased risk of several other metabolic disorders such as cardiovascular diseases, renal diseases, inflammation and mental health conditions such as depression [3–6]. Prevention of the onset of pre-diabetes and progression of pre-diabetes to T2D are therefore of utmost importance to improve public health. It is widely accepted that unhealthy dietary habits, physical inactivity and obesity are risk factors for T2D in addition to genetics and family history of T2D [7]. Therefore, modifying lifestyle factors such as diet is an effective strategy to reduce the risk of T2D. One way to achieve this may be by adopting a diet, which contributes to a slow increase in glycaemic response (GR) and insulinaemic response (IR) [8].

Several factors are known to affect GR such as the amount and type of carbohydrates in foods, presence of other food components such as fat, protein and antinutrients, food processing methods and food structure [9]. Whilst adding fat or protein to high carbohydrate foods are both effective in lowering GR of foods, protein has been shown to be two to three times more effective in reducing GR compared to fat [10]. The effect of whey protein on attenuating postprandial GR with concomitant increase in IR has been demonstrated in previous studies [11,12]. More recently, Stevenson and Allerton [13] reviewed the literature on acute and long-term intervention studies using whey protein in different doses and with different meals and study designs, indicating potential detrimental effects of increased IR on longer-term insulin sensitivity. A cause-and-effect relationship has been highlighted between hyperinsulinaemia and inflammation leading to various metabolic diseases such as obesity, T2DM and cancers [14,15]. Moreover, higher protein intake has also been shown to increase postprandial blood glucose response in Type 1 Diabetes, thereby requiring higher doses of insulin for glycaemic control [16]. It is therefore pertinent to identify alternative protein sources that are capable of alleviating hyperinsulinaemia whilst lowering GR.

With the current interest in plant-based diets as sustainable food sources for improving physical and mental health [17,18], various plant protein isolates have been used in GR research. Mixed meals or isolates based on soy, potato, rice, oat and pea were used in previous studies [19–21]. Pea protein from yellow pea (*Pisum sativum*) is gaining popularity as a sustainable alternative to animal proteins in various food product development applications [22]. However, Re et al [23] did not find any difference in GR to soup samples with 15 and 30 g of pea protein and 30 g whey protein. On the other hand, IR was lower when 30 g pea protein and 30 g whey protein were compared with 15 g pea protein, potentially due to the different carbohydrate contents of the test meals. Similarly, a single dose (24 g) of oat, pea and rice proteins in chocolate beverages did not demonstrate significant differences in GR; but a higher IR was induced by oat and pea protein in comparison with rice protein [20]. In a recent study using pea protein (25 g and 50 g) consumed with isoglucidic drinks containing 50 g glucose, a dose-dependent decrease in GR and increase in IR were reported [24]. According to a systematic review by Lonnie et al. [25], pea is one of the most used plant protein sources to study postprandial GR. However, lack of homogeneity in doses, comparators and forms used in studies made a direct comparison of results impossible in that review.

Whilst diet alone may not prevent chronic diseases, dietary components such as proteins contribute to various metabolic effects such as increased energy expenditure, gluconeogenesis, ketogenesis and satiety by preserving fat free mass. Weight loss effects of high protein diets may therefore help to prevent obesity and associated metabolic diseases [26]. In a study comparing 50 g/day whey protein and pea protein, both were found to be equally effective in increasing muscle thickness in male participants [27]. However, evidence on metabolic effects of pea protein and other novel plant proteins is still evolving. Considering the average recommended protein intake levels for men (56g/day) and women (45 g/day) [28], it is realistic to consume a 15–20 g protein per portion/meal. In this context, it is logical to compare the dose response effects of different protein supplements at this lower intake levels matching with commercially available products such as protein bars and shakes [29] to determine their effects on postprandial IR. Therefore, the aim of this study was to compare the GR and IR to 10g NUTRALYS^®^ S85 Plus pea protein, 20g NUTRALYS^®^ S85 Plus pea protein, 10g whey protein concentrate and 20g whey protein concentrate co-ingested with 50 g glucose.

## 2. Materials and methods

### 2.1. Test foods

The reference product used was Glucose (Myprotein, UK) with 91% available carbohydrate. The test products used were glucose with 10 g and 20 g NUTRALYS^®^ S85 Plus pea protein (GLU + 10g-PP or GLU + 20g-PP; Roquette Freres, Lestrem, France) and glucose with 10 g and 20 g whey protein concentrate (GLU + 10g-WP or GLU + 20g-WP; Friesland Campina, Amersfoort, The Netherlands). The reference product GLU (50 g glucose) was compared with GLU + 10g-PP, GLU + 20g-PP, GLU + 10g-WP and GLU + 20g-WP. Table 1 shows the nutrition information of the products. All the products were consumed as beverages by dissolving the powders in 250 ml water.

**Table 1:** Nutrition information and amino acid profile of GLU, GLU + 10g-PP, GLU + 20g-PP, GLU + 10g-WP and GLU + 20g-WP consumed in the study.

	GLU	GLU + 10g-PP	GLU + 20g-PP	GLU + 10g-WP	GLU + 20g-WP
Energy(kCal/kJ)	200/842.5	241/1015.2	280.2/1180.3	237.8/1001.7	275.6/1161
Available Carbohydrates (g)	50	50	50	50	50
Protein (g)	0	8	16	8.1	16.2
Fat (g)	0	0.9	1.8	0.6	1.2
Dietary Fibre (g)	0	0.1	0.2	0	0
**Amino acid profile (g)**					
Aspartic acid	0	1.15	2.3	1.18	2.36
Glutamic acid	0	1.67	3.34	1.83	3.66
Alanine	0	0.43	0.86	0.51	1.02
Arginine	0	0.87	1.74	0.31	0.62
Cysteine	0	0.1	0.2	0.28	0.56
Glycine	0	0.4	0.8	0.19	0.38
Histidine	0	0.25	0.5	0.22	0.44
Isoleucine	0	0.47	0.94	0.57	1.14
Leucine	0	0.82	1.64	1.29	2.58
Lysine	0	0.71	1.42	1.07	2.14
Methionine	0	0.11	0.22	0.24	0.48
Phenyl alanine	0	0.55	1.1	0.39	0.78
Proline	0	0.43	0.86	0.51	1.02
Serine	0	0.51	1.02	0.47	0.94
Threonine	0	0.38	0.76	0.54	1.08
Tyrosine	0	0.38	0.76	0.37	0.74
Valine	0	0.5	1	0.53	1.06
Tryptophan	0	0.1	0.2	0.21	0.42

GLU (Glucose), GLU + 10g-PP (Glucose + 10g NUTRALYS^®^ S85 Plus Pea Protein), GLU + 20g-PP (Glucose + 20g NUTRALYS^®^ S85 Plus Pea Protein), GLU + 10g-WP (Glucose + 10g Whey Protein Concentrate) and GLU + 20g-WP (Glucose + 20g Whey Protein Concentrate).

### 2.2. Participants

The study was approved by the University Research Ethics Committee (UREC) at Oxford Brookes University (UREC Registration No: 140806, 110594 and 211543). Recruitment of participants was carried out through posters, announcements in lectures, personal networks and via social media. All participants were given the opportunity to review the study protocol and ask questions prior to giving written informed consent to take part in the study.

Forty-four healthy, moderately active, non-smokers were recruited (17 male, 27 female; aged 19 to 57 years) after assessing their eligibility using a health questionnaire: The inclusion criteria were:

- Age 18 to 60 years
- Not pregnant or lactating
- Body mass index (BMI) ≤30 kg/m^2^
- Fasting blood glucose value < 6.1 mmol/l
- No diabetes or impaired glucose tolerance
- No known food allergy or intolerance
- No medical condition(s) or medication(s) known to affect glucose regulation or appetite and/or which influence digestion and absorption of nutrients
- No known history of diabetes mellitus or the use of antihyperglycaemic drugs or insulin to treat diabetes and related conditions
- No major medical or surgical event requiring hospitalization within the preceding 3 months
- Not using steroids, protease inhibitors or antipsychotics (all of which have major effects on glucose metabolism and body fat distribution).

### 2.3. Anthropometric measurements

Height of the participants was measured to the nearest centimetre using a stadiometer (Seca Ltd, Birmingham, UK). A Tanita MC-980 MA body composition analyser (Tanita UK Ltd, Manchester, UK) was used to record, body weight, fat mass and lean body mass. Body mass index (BMI) was calculated using the formula: weight (kg)/height (m)^2^. All anthropometric measurements were conducted after overnight fasting, with participants wearing light clothing and no shoes.

### 2.4. Study design and protocol

The GR and IR to GLU, GLU + 10g-PP, GLU + 20g-PP, GLU + 10g-WP and GLU + 20g-WP were tested using a single-blind, randomised, repeated measures crossover trial design (S1 File Study Protocol). The participants were randomly assigned by the researchers at Oxford Brookes Centre for Nutrition and Health (OxBCNH) to test the reference or test products by simple randomization using a pseudo-random number generator [30]. Sequential numbering of 35 sets with 5 numbers per set was used to implement the random allocation. The researchers who enrolled and assigned the participant to the intervention had access to the random allocation sequence. The trial was registered with Clinical Trials.gov (https://clinicaltrials.gov/; NCT04871971) on 3^rd^ May 2021 and conducted at OxBCNH at Oxford Brookes University between September 2021 and July 2022. The trial protocol and statistical analysis plan can be accessed on the clinicaltrials.gov website. Participant recruitment started on 15^th^ September 2021 and ended on 18^th^ July 2022.

The protocol used was in accordance with ISO 26642 standards [31] and there were no changes to the trial after it commenced. On the day prior to the test session, participants were asked to limit alcohol, caffeinated drinks and intense physical activity. They were also told to standardise their diet and physical activity for 24 hours before each test and to fast after 21:00 the night before a test, with only water allowed, in moderation. After a 12-hour overnight fast, the test session started before 10:00 and the participants were given 15 minutes to consume the test products. Each participant tested the products on separate days in random order, maintaining at least five-days between two products. The participants were blinded to the products, which were all served as glucose drinks with the respective protein doses dissolved in them.

### 2.5. Blood glucose and insulin measurements

Participants warmed their hands to encourage blood flow before finger-prick blood sampling using a Unistik^®^3 single-use lancing device (Owen Mumford, Woodstock, UK). Baseline capillary blood glucose value was recorded as the average of measurements at −5 min and 0 min before consuming the products. After starting to drink the products, additional capillary blood samples were taken every 15 minutes in the first hour and every 30 minutes in the second and third hours. Plasma insulin values were also recorded at each test time point (−5, 0, 15, 30, 45, 60, 90, 120, 150 and 180 minutes) during the three-hour test session. For this, 400–500 μL of capillary blood was obtained by finger pricks and blood was collected into chilled microvette^®^ capillary blood collection tubes treated with Dipotassium EDTA (CB 300 K2E; Sarstedt Ltd, Numbrecht, Germany). The HemoCue Glucose 201 DM analyser (HemoCue^®^ Ltd, Ängelholm, Sweden) was used to measure blood glucose (mmol/L). Plasma (150 μL) was obtained by centrifuging the microvette^®^ tubes and insulin concentration (μU/ml) in the plasma samples was determined by electrochemiluminescence immunoassay using an automated analyzer (Cobas^®^ E411; Roche diagnostics, Vienna, Austria). The blood glucose and plasma insulin incremental area under the curve (iAUC) for both the reference and test products were calculated geometrically by applying the trapezoid rule [31].

### 2.6. Statistical analyses

A previous GR study using pea protein in healthy individuals was used to determine sample size [23]. A sample size of 30 participants was necessary to detect a 72.7 mmol/l*min (SD 76.2) reduction in postprandial glucose iAUC to demonstrate 90% power with a two-sided α-level of 5%. Therefore, 44 participants were recruited in this study, accounting for any attrition. There were no adverse or serious adverse events in this study.

Data was analysed using the IBM Statistical Package for the Social Sciences (SPSS) version 25 (SPSS Inc., Chicago, Illinois). Prior to statistical analysis, the normality of the data was tested using the Shapiro-Wilks statistic. For change in blood glucose and plasma insulin from baseline and iAUC for blood glucose and plasma insulin, the main effects of test products and time and their interaction effects were determined by using a two-way repeated measures analysis of variance (RM ANOVA). The Greenhouse-Geisser correction was used where sphericity was violated. Where significant test product x time interactions were detected, pairwise comparisons were made between the test products with a simple effects analysis using the Sidak correction. For blood glucose and plasma insulin peak concentrations and time of the peak concentrations, a one-way repeated measures ANOVA (for normally distributed data) or a non-parametric Friedman test (where data were not normally distributed) was used. Post hoc analyses were performed using the Bonferroni correction for normally distributed data and the Wilcoxon signed-rank test for non-normal data. Data are presented as mean, standard deviation (SD) and standard error of the mean (SEM) values. Statistical significance was set at p < 0.05 for all tests, with the exception of Wilcoxon signed-rank tests (where required), which was conducted with a Bonferroni correction applied, resulting in a significance level set at p < 0.005. Data from participants who withdrew during the study were deleted and excluded from analysis. Data from all randomized participants who completed all the test sessions were included in the analysis. The individual de-identified data and statistical code can be made available on demand by contacting OxBCNH. The data is not publicly available due to intellectual property rights.

## 3. Results

**Fig 1:**
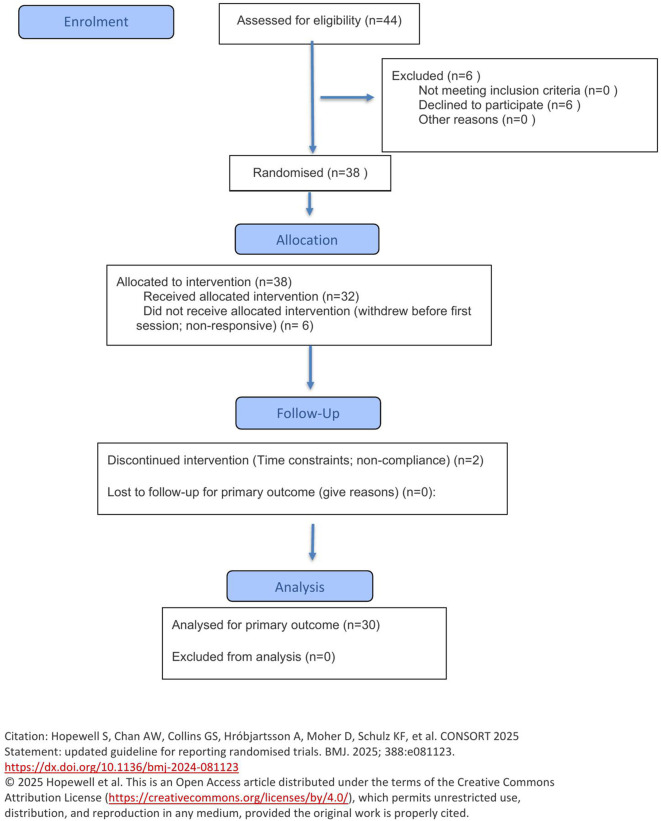
CONSORT 2025 flow diagram of the progress through the phases of the randomised crossover trial of one group.

Out of the forty-four participants recruited, six withdrew from the study prior to the first test session and a further eight were nonresponsive or withdrew from the study due to time constraints or failure to comply with the experimental procedures. Therefore, the results reported are for thirty participants (13 Male, 17 Female). Fig 1 shows the Consort flow diagram of the progress through the phases of the randomised crossover trial (S2 File CONSORT Checklist). The physical characteristics of the included study population are presented in Table 2.

**Table 2:** Physical characteristics of the included study population (mean ± SD).

	All participants (*n* 30)
Age (y)	31.1 ± 10.0
Height (m)	1.7 ± 0.1
Weight (kg)	68.1 ± 12.5
BMI (kg/m^2^)	23.2 ± 2.8
Fat mass (%)	24.1 ± 7.7
Lean body mass (kg)	51.7 ± 11.0

SD, standard deviation.

### 3.1. Glycaemic response

**Fig 2:**
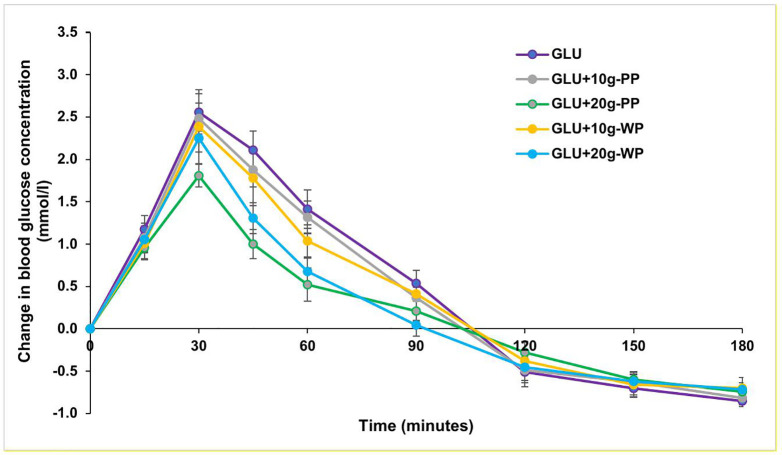
GR curves for GLU, GLU + 10g-PP, GLU + 20g-PP, GLU + 10g-WP and GLU + 20g-WP. Data are presented as mean and SEM (n 30). GLU (Glucose), GLU + 10g-PP (Glucose + 10g NUTRALYS^®^ S85 Plus Pea Protein), GLU + 20g-PP (Glucose + 20g NUTRALYS^®^ S85 Plus Pea Protein), GLU + 10g-WP (Glucose + 10g Whey Protein Concentrate) and GLU + 20g-WP (Glucose + 20g Whey Protein Concentrate). SEM, standard error of the mean.

There was a significant main effect of test products (*F*(4,116)=8.01, *p* < 0.001, η^2^_p_=0.22) and time (*F*(3,84)=102.43, *p* < 0.001, η^2^_p_=0.78) on change in blood glucose. There was also a significant interaction between test products and time (*F*(11,328)=4.88, *p* < 0.001, η^2^_p_=0.14). As shown in Fig 2, the change in blood glucose from baseline was significantly lower for GLU + 20-PP compared with GLU at 30 (*p* = 0.011, mean difference = −0.75, 95% CI=[−1.4,-.12]), 45 (*p* < 0.001, mean difference = −1.11, 95% CI=[−1.7,-.51]) and 60 (*p* < 0.001, mean difference = −0.89, 95% CI=[−1.5,-.33]) minutes. GLU + 20-PP resulted in lower blood glucose response compared to GLU + 10-PP at 30 (*p* = 0.026, mean difference = −0.68, 95% CI=[−1.3,-.05]), 45 (*p* = 0.001, mean difference = −0.88, 95% CI=[−1.5,-.3]) and 60 (*p* = 0.002, mean difference = −0.8, 95% CI=[−1.4,-.22]) minutes. Similarly, GLU + 20-WP elicited lower blood glucose response compared to GLU + 10-PP at 45 (*p* = 0.011, mean difference = −0.57, 95% CI=[−1.05,-.09]) and 60 (*p* = 0.001, mean difference = −0.64, 95% CI=[−1.05,-.23]) minutes. The incremental blood glucose response to GLU + 20-PP was significantly lower than GLU + 10-WP at 30 (*p* = 0.002, mean difference = −0.58, 95% CI=[.17,.99]) and 45 (*p* = 0.001, mean difference = −0.78, 95% CI=[−1.29,-.28]) minutes. Finally, the change in blood glucose after GLU + 20-WP was significantly lower compared to GLU + 10-WP at 45 minutes (*p* = 0.038 mean difference = −0.47, 95% CI=[−.93,-.02]).

Table 3 shows the blood glucose iAUC, peak blood glucose and time of peak glucose for the five test drinks. There was a significant main effect of test products (*F*(4,116)=11.57, *p* < 0.001, η^2^_p_=0.29) and time (*F*(1.16,33.6)=44.4, *p* < 0.001, η^2^_p_=0.61) on the mean blood glucose iAUC. There was also a significant interaction between test products and time (*F*(3.5,102.6)=3.54, *p* = 0.013, η^2^_p_=0.14). Pairwise comparisons revealed a significantly lower mean iAUC for GLU + 20g-PP compared to GLU at 60 (*p* < 0.001, mean difference = −36.5, 95% CI=[−57.5,-15.6]), 90 (*p* < 0.001, mean difference = −52.1, 95% CI=[−80.5,-23.6]), 120 (*p* < 0.001, mean difference = −55.2, 95% CI=[−88.5,-21.9]) and at 180 (*p* = 0.003, mean difference = −53.4, 95% CI=[−92.4,-14.4]) minutes. Similarly, the iAUC for GLU + 20g-PP was significantly lower than GLU + 10g-WP at 60 (*p* = 0.001, mean difference = −29.7, 95% CI=[−50.2,-9.3]), 90 (*p* < 0.001, mean difference = −41.3, 95% CI=[−67,-15.5]), 120 (*p* = 0.001, mean difference = −42.2, 95% CI=[−71.5,-12.9]) and 180 (*p* = 0.01, mean difference = −40.2, 95% CI=[−73.3,-7.1]) minutes. The iAUC for GLU + 20g-WP was also significantly lower than that of GLU at 60 (p = 0.006, mean difference = −23.9, 95% CI=[−42.8,-5]), 90 (p < 0.001, mean difference = −40.3, 95% CI=[−65.4,-15.1]), 120 (p = 0.001, mean difference = −45.4, 95% CI=[−75.8,-15]) and 180 minutes (p = 0.005, mean difference = −44.7, 95% CI=[−79.5,-9.9]). GLU + 20g-WP had a significantly lower iAUC than GLU + 10g-PP at 60 (p = 0.038, mean difference = −17.1, 95% CI=[−33.7,-.6]), 90 (p = 0.002, mean difference = −29.4, 95% CI=[−50.3,-8.6]), 120 (p = 0.002, mean difference = −32.4, 95% CI=[−54.9,-9.8]) and 180 (p = 0.003, mean difference = −31.4, 95% CI=[−54.3,-8.6]) minutes.

**Table 3:** Mean (± SD) iAUC blood glucose (mmol/l*min) at 60, 90, 120 and 180 minutes after consumption of GLU, GLU + 10g-PP, GLU + 20g-PP, GLU + 10g-WP and GLU + 20g-WP.

iAUC	GLU	GLU + 10g-PP	GLU + 20g-PP	GLU + 10g-WP	GLU + 20g-WP	*P* value
iAUC 60	99.1 ± 47.4^a^	89.4 ± 37.9^ab^	62.6 ± 28.1^bd^	86.2 ± 34.6^acd^	75.2 ± 35.8^d^	<0.001*
iAUC 90	129.7 ± 65.1^a^	118.9 ± 53.7^ab^	77.6 ± 40.6^bd^	108.9 ± 46.3^acd^	89.5 ± 48.2^d^	<0.001*
iAUC 120	140.3 ± 70.6^a^	127.2 ± 63.8^ab^	85.1 ± 46.8^bd^	116.9 ± 51.2^acd^	94.9 ± 53.5^d^	<0.001*
iAUC 180	143.2 ± 74.0^a^	131.3 ± 68.4^ab^	89.8 ± 51.6^bd^	120.3 ± 53.4^acd^	98.5 ± 58.0^d^	<0.001*
Peak blood glucose (mmol/l)	7.6 ± 1.2 ^a^	7.6 ± 0.9 ^a^	6.9 ± 0.7 ^b^	7.3 ± 0.8	7.2 ± 0.8	<0.001*
Time of peak blood glucose (min)	34.5 ± 10.5	34.0 + 10.4	32.0 ± 11.0	35.5 ± 18.7	31.0 ± 7.8	0.408

*Statistically significant difference (*p* < 0.05); SD, standard deviation. Values with different superscripts in each row are significantly different from each other. GLU (Glucose), GLU + 10g-PP (Glucose + 10g NUTRALYS^®^ S85 Plus Pea Protein), GLU + 20g-PP (Glucose + 20g NUTRALYS^®^ S85 Plus Pea Protein), GLU + 10g-WP (Glucose + 10g Whey Protein Concentrate) and GLU + 20g-WP (Glucose + 20g Whey Protein Concentrate).

There was a significant difference in the mean peak blood glucose between all five test drinks (F(4, 116) = 7.020, p < 0.001). However, there was no significant difference (x^2^(4) = 3.986, p = 0.408) in the time of the blood glucose peak (Table 3). Post hoc tests revealed that the mean peak blood glucose for GLU + 20g-PP was significantly lower than that of GLU (p = 0.008) and GLU + 10g-PP (p = 0.002). No other significant differences were observed.

### 3.2. Insulinaemic response

**Fig 3:**
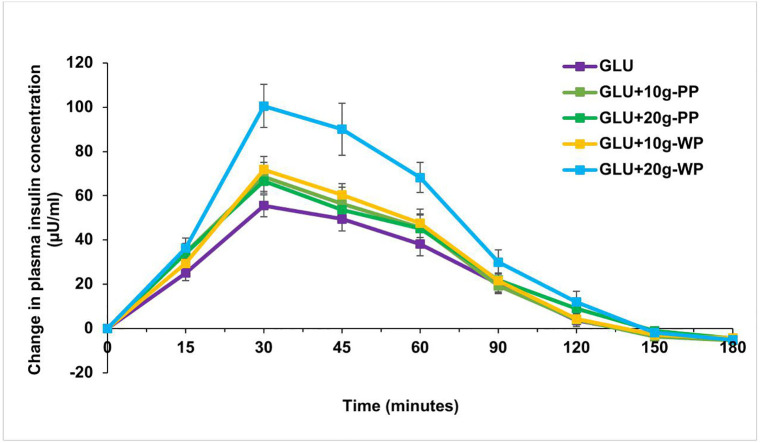
IR curves for GLU, GLU + 10g-PP, GLU + 20g-PP, GLU + 10g-WP and GLU + 20g-WP. Data are presented as mean and SEM (n 30). GLU (Glucose), GLU + 10g-PP (Glucose + 10g NUTRALYS^®^ S85 Plus Pea Protein), GLU + 20g-PP (Glucose + 20g NUTRALYS^®^ S85 Plus Pea Protein), GLU + 10g-WP (Glucose + 10g Whey Protein Concentrate) and GLU + 20g-WP (Glucose + 20g Whey Protein Concentrate). SEM, standard error of the mean.

There was a significant main effect of test products (*F*(2.9,80)=13.8, *p* < 0.001, η^2^_p_=0.33) and time (*F*(2.5,70)=85.1, *p* < 0.001, η^2^_p_=0.75) on change in plasma insulin. There was also a significant interaction between test products and time (*F*(5.8,161.4)=4.2, *p* = 0.001, η^2^_p_=0.13). As illustrated in Fig 3, the change in plasma insulin from baseline for GLU + 10g-WP was significantly higher than GLU (p = 0.022, mean difference = 15.3, 95% CI=[1.5,29.2]) at 30 minutes. GLU + 20g-WP induced a significantly higher change in plasma insulin from baseline when compared to GLU at 30 (p < 0.001, mean difference = 40.8, 95% CI=[17.6,64]), 45 (p = 0.007, mean difference = 41.5, 95% CI=[8.5,74.5]) and 60 minutes (p < 0.001, mean difference = 30.9, 95% CI=[14.3,47.6]). Similarly, the change in plasma insulin for GLU + 20g-WP was significantly higher than GLU + 10g-PP at 30 (p = 0.012, mean difference = 28.1, 95% CI=[4.3,51.9]), 45 (p = 0.007, mean difference = 34.5, 95% CI=[7,62]) and 60 minutes. (p = 0.003, mean difference = 22.9, 95% CI=[5.8,40]. Lastly, GLU + 20g-WP also induced a significantly higher change in plasma insulin from baseline when compared to GLU + 20g-PP at 45 (p = 0.002, mean difference = 37.9, 95% CI=[11.3,64.5]) and 60 (p = 0.011, mean difference = 24.9, 95% CI=[4,45.8]) minutes.

Table 4 shows the plasma insulin iAUC for the 5 test drinks. There was a significant main effect of test products (*F*(2.9,82.7)=15, *p* < 0.001, η^2^_p_=0.34) and time (*F*(1.1,32.7)=72.8, *p* < 0.001, η^2^_p_=0.72) on the mean plasma insulin iAUC. There was also a significant interaction between test products and time (*F*(3.1,90.2)=4.3, *p* = 0.006, η^2^_p_=0.13). Pairwise comparisons revealed that GLU + 20g-WP had a significantly higher mean insulin iAUC compared to GLU at 60 (p < 0.001, mean difference = 1680.6, 95% CI=[816,2545]), 90 (p < 0.001, mean difference = 2276.6, 95% CI=[1177.7,3375.5]), 120 (p < 0.001, mean difference = 2558.5, 95% CI=[1227.2,3889.8]) and 180 (p < 0.001, mean difference = 2695.5, 95% CI=[1165.5,4225.6]) minutes. GLU + 20g-WP induced a significantly higher mean insulin iAUC compared to GLU + 10g-PP at 60 (p < 0.001, mean difference = 1198, 95% CI=[445,1950.7]), 90 (p < 0.001, mean difference = 1694, 95% CI=[628.6,2758.8]), 120 (p = 0.002, mean difference = 1973.4, 95% CI=[554.8,3392]) and 180 (p = 0.008, mean difference = 2116.6, 95% CI=[413.7,3819.5]) minutes. Insulin iAUC for GLU + 20g-WP was significantly higher than GLU + 20g-PP at 60 (p < 0.001, mean difference = 1288.7, 95% CI=[514.4,2063.1]), 90 (p < 0.001, mean difference = 1753.9, 95% CI=[851,2656.7]), 120 (p < 0.001, mean difference = 1938.2, 95% CI=[867,3009.5]) and 180 (p < 0.001, mean difference = 2007.3, 95% CI=[808.7,3205.9]) minutes. Similarly, the insulin iAUC for GLU + 20g-WP was significantly higher than GLU + 10g-WP at 60 (p = 0.007, mean difference = 1162, 95% CI=[232.1,2092.1]), 90 (p = 0.001, mean difference = 1596.6, 95% CI=[504.7,2688.6]), 120 (p = 0.001, mean difference = 1859.8, 95% CI=[585.4,3134.3]), and 180 minutes (p = 0.004, mean difference = 1949.6, 95% CI=[473.2,3426]).

**Table 4:** Mean (± SD) iAUC plasma insulin (µU/ml*min) at 60, 90, 120 and 180 minutes after consumption of GLU, GLU + 10g-PP, GLU + 20g-PP, GLU + 10g-WP and GLU + 20g-WP.

iAUC	GLU	GLU + 10g-PP	GLU + 20g-PP	GLU + 10g-WP	GLU + 20g-WP	*P* value
iAUC 60	2234.8 ± 1112.2 ^a^	2717.4 ± 1449.8 ^a^	2626.6 ± 920.3 ^a^	2753.3 ± 1163.4 ^a^	3915.35 ± 1837.0 ^b^	<0.001*
iAUC 90	3110.0 ± 1594.5 ^a^	3692.9 ± 1946.0 ^a^	3632.7 ± 1377.2 ^a^	3789.9 ± 1586.5 ^a^	5386.54 ± 2550.7 ^b^	<0.001*
iAUC 120	3373.4 ± 1864.5 ^a^	4069.7 ± 2182.4 ^a^	4104.9 ± 1679.9 ^a^	4183.3 ± 1818.5 ^a^	6043.07 ± 3114.0 ^b^	<0.001*
iAUC 180	3617.6 ± 1918.1 ^a^	4195.2 ± 2317.1 ^a^	4304.6 ± 1896.1 ^a^	4362.2 ± 1929.0 ^a^	6311.81 ± 3489.1 ^b^	<0.001*
Peak plasma insulin (µU/ml)	73.43 ± 31.16^a^	88.70 ± 39.28	90.17 ± 35.84^b^	91.64 ± 36.20^c^	127.71 ± 68.42^d^	p < 0.001*
Time of peak plasma insulin (min)	38.00 ± 14.06	39.00 ± 12.21	35.00 ± 11.37	38.50 ± 12.88	38.50 ± 12.26	p = 0.681

*Statistically significant difference (*p* < 0.05); SD, standard deviation. Values with different superscripts in each row are significantly different from each other. GLU (Glucose), GLU + 10g-PP (Glucose + 10g NUTRALYS^®^ S85 Plus Pea Protein), GLU + 20g-PP (Glucose + 20g NUTRALYS^®^ S85 Plus Pea Protein), GLU + 10g-WP (Glucose + 10g Whey Protein Concentrate) and GLU + 20g-WP (Glucose + 20g Whey Protein Concentrate).

There was a significant difference in the mean peak plasma insulin concentration between all five test drinks (x^2^(4) = 45.322, p < 0.001). However, there was no significant difference in the time of plasma insulin peak (Table 4). Post hoc tests revealed that GLU had a significantly lower insulin peak compared to GLU + 20g-PP (p = 0.001), GLU + 10g-WP (p < 0.001) and GLU + 20g-WP (p < 0.001). GLU + 20g-WP had a significantly higher insulin peak than GLU, GLU + 10g-PP, GLU + 20g-PP and GLU + 10g-WP (p < 0.001). No other significant differences were observed.

## 4. Discussion

This study aimed to determine the dose-dependent effects of whey protein and pea protein on GR and IR of a glucose drink in healthy human participants. The findings of this study showed that both proteins are effective in lowering GR with concurrent increases in IR. However, the magnitude of increase in insulin iAUC was approximately 32% lower for GLU + 20g-PP in comparison with GLU + 20g-WP. Irrespective of this, GLU + 20g-PP resulted in a greater reduction in GR compared to GLU + 20g-WP. According to a recent systematic review, the effect of plant protein on postprandial GR was equivalent to animal protein, with no superior effect [25]. Therefore, to the best of our knowledge, a superior effect of pea protein over whey protein on IR is demonstrated for the first time in this study. This is of relevance in food product or supplement development, considering the acute insulinotropic effect of whey protein reported in previous studies [21,32,33]. Moreover, such products may also be useful for nutritional therapy in T2DM to control hyperinsulinaemia or in Type 1 Diabetes where there is protein-induced increase in insulin demand [16]. With the current increased interest in environmentally sustainable protein sources among food product manufacturers, there is evidence that pea protein-based products may have lower greenhouse gas emissions than animal-based products [34].

When co-ingested with glucose, whey protein lowers GR by increasing insulin secretion mediated by three mechanisms – augmented incretin hormones (Glucagon-like-peptide-1 (GLP-1) and Glucose-dependent insulinotropic polypeptide (GIP)) production, delayed gastric emptying and inhibition of Dipeptidyl Peptidase-IV (DPP-IV) that breaks down the incretin hormones [35,36]. Whilst the interrelation between these processes presents a challenge in clearly understanding the exact mechanism involved, this discussion attempts to unravel any differences in the effect of pea protein on the aforementioned responses.

In this study, 10 g and 20 g of whey protein and pea protein were used with a 50 g glucose beverage. As expected, both GLU + 20g-PP and GLU + 20g-WP lowered GR compared to the reference product GLU. However, GLU + 20g-PP elicited the most significant reduction of 37% in glucose iAUC over three hours compared with a 30% reduction after GLU + 20g-WP consumption. Furthermore, the peak blood glucose following GLU + 20g-PP was 0.7 mmol/L lower compared to GLU, whereas with GLU + 20g-WP, a reduction of 0.4 mmol/L was observed. The results for whey protein are consistent with previous studies where a significant reduction in GR was reported following ingestion of higher doses of whey protein (30 g to 45 g) mixed with different low and high sugar foods and beverages [21,23,37]. It is well known that this effect is mediated to some degree by increased circulating insulin concentration activated by branched chain amino acids (BCAAs; leucine, isoleucine, valine) and other essential amino acids (lysine, threonine, tryptophan) present in higher proportions in whey protein [23,37]. However, a positive association between high consumption of BCAAs and increased risk of T2D has been noted in epidemiological studies [38] supported with further evidence of improved insulin sensitivity following reduction of BCAA intake in a randomised controlled trial [39]. Pea protein has demonstrated hypoglycaemic effects when used with low and high sugar foods/drinks in previous studies, at doses ranging from 15 g to 50 g [23,24,40]. Some of the mechanisms of glucose lowering may be similar to that of whey protein as reported by Smith et al. [40] involving increase in plasma amino acids that accelerate plasma insulin secretion. However, considering the levels of BCAAs in pea protein [23], other amino acids such as glycine, phenylalanine and arginine may also be involved in moderately accentuating IR [41,42]. Moreover, higher glycine and arginine levels may improve insulin sensitivity and reduce the risk of T2D [43,44]. This controlled increase in IR may be beneficial for alleviating potential longer-term negative effects on insulin sensitivity with prolonged pea protein intake. The disparity in IR to pea and whey proteins co-ingested with glucose may be explained by differences in the amino acid composition, bioactive peptides released and consequent expression of peptide and glucose transporters at the enterocyte level [45]. As a result, pea protein may have the potential to reduce intestinal glucose uptake, mitigating an uncontrolled increase in postprandial insulinaemia.

As reported previously [35], the IR for pure glucose was lower than that following the ingestion of glucose with protein, in this study. However, the aim of this study was to identify a protein source that decreases GR without disproportionately increasing IR. To that effect, IR to GLU + 20g-PP was markedly diminished compared to GLU + 20g-WP. In addition to that, the peak plasma insulin concentration was 29% lower after GLU + 20g-PP than following GLU + 20g-WP intake. The insulinogenic effect following whey protein intake occurs by an enhanced expression of G-protein coupled receptors in the pancreatic islet β-cells, mediated by the secretion of the incretin hormones - GLP-1 and GIP – from the gut [35,46]. This incretin effect may also be assisted by the Dipeptidyl Peptidase-IV (DPP-IV) inhibition by whey protein derivatives [35,36].

The role of GLP-1 and GIP in pea protein mediated effects is inconclusive. Despite trends similar to IR, previous studies with higher doses of pea protein (20 g to 50 g) did not contribute to any significant increase in GLP-1 or GIP iAUC [20,23,24] This may suggest a weak DPP-IV inhibitory effect of pea protein compared to whey protein. Conflicting this hypothesis, *in vitro* and animal experiments [47] have concluded that pea protein is a potent DPP-IV inhibitor, although hydrolysed forms of pea protein used in the *in vitro* and *ex vivo* studies might explain the result. Moreover, whey protein administration was not possible in the animal study due to methodological constraints [48], thereby making a direct comparison of DPP-IV inhibition by the two proteins impossible. It is also worth noting that pure protein solution was provided in animal and *in vitro* studies [48] as opposed to the current study where pea protein and whey protein were co-ingested with a glucose solution. According to Mignone et al. [35], GIP and GLP-1-mediated insulinotropic effects are dependent on elevated glucose levels. Furthermore, *in vitro* and *ex vivo* studies cannot replicate the *in vivo* glucose-homeostasis mechanisms such as digestion kinetics and expression of peptide and glucose transporters completely [45,47] and for this reason, further human studies are warranted to evaluate the DPP-IV inhibition potential of pea protein.

NUTRALYS^®^ S85 Plus pea protein used in this study is characterised by fast digestibility [49], similar to whey protein which is considered as a fast-digesting protein [35]. However, minor differences in viscosity and digestibility profile may have influenced the rate of incretin hormone secretion and DPP-IV inhibition, consequently leading to a moderate increase in IR in this study. This assumption is supported by Overduin et al. [50] who reported that an intermediate-fast pea protein formed smaller aggregates during gastric digestion in rats, resulting in delayed intestinal bioavailability compared to whey protein.

Delayed gastric emptying, which is linked to GLP-1 and the interaction between the nutrients in the small intestine, is another mechanism known to lower postprandial glucose concentrations following intake of glucose and whey protein [35,51]. The intermediate digestibility of pea protein may have contributed to a slight delay in gastric emptying; however, diminished GLP-1 secretion following pea protein intake may have weakened this effect [20,23,24,50]. Further studies are warranted to compare the differential impact of whey protein and pea protein on the aforementioned multiple mechanisms involved in reducing GR.

Whilst this research has demonstrated the beneficial effect of pea protein on reducing GR with a moderate increase in IR, there are some limitations. Only one form of extracted pea protein isolate and whey protein (concentrate) were tested in this trial. Therefore, the results cannot be generalised to the different forms of protein such as isolates, hydrolysates and concentrates based on processing methods used, which may show different effects based on their digestibility and bioavailability [25]. Although all test products were sweetened with glucose, the taste differences between pea protein and whey protein might have resulted in participants perceiving the sensory differences in samples. Additionally, it was not possible to measure incretin hormones or digestion kinetics in this study to comprehensively explain the differences observed. Despite these limitations, this study was able to identify significant differences between pea protein and whey protein for GR and IR, demonstrating the robustness of our findings. It is recognised that the results should be interpreted with caution, however, they provide valuable insights and contribute meaningfully to the existing body of knowledge on metabolic effects of dietary proteins. Likewise, the study of other amino acid profiles on insulin resistance by combining milk protein or other sources of plant-based proteins with pea protein, would be interesting to better understand the mechanism of action. A pure glucose drink was used with the test proteins, which does not compare with composite meals where macronutrients may have an interactive influence on GR and IR and their underlying mechanisms discussed above. Lastly, the production of pea protein isolate may have a cost impact as in any other protein isolate manufacturing.

## 5. Conclusions

In conclusion, this study has shown a superior effect of pea protein over whey protein in reducing postprandial GR, when consumed with a 50 g glucose beverage, without any excessive increase in IR. Postprandial GR was reduced in a dose dependent manner using both pea protein and whey protein. However, the insulinogenic effect of pea protein was significantly lower than that of whey protein at 20 g dose. This may be due to the dissimilar amino acid profile, the different rheological comportment and/or the liberation of bioactive peptides during digestion of pea protein compared to whey protein acting differently on incretin hormone production, DPP-IV inhibition or intestinal glucose absorption Therefore, pea protein may prove to be an effective and environmentally sustainable alternative to whey protein in reducing GR and IR of a refined carbohydrate.

## Supporting information

S1 File Study Protocol for glycaemic response and insulinaemic response study. (PDF)

S2 File CONSORT checklist for the randomised controlled trial. (PDF)
