# Supplementary material for: A randomized controlled trial in healthy participants to compare the insulinogenic effects of whey protein and pea protein co-ingested with glucose
Source: PLoS One. 2026 Jan 30;21(1):e0340386. doi: 10.1371/journal.pone.0340386 (PMC12857942; doi:10.1371/journal.pone.0340386)
Supplement: S1 File — (PDF) [file pone.0340386.s001.pdf]

S1 Study Protocol
Protocol for glycaemic response and insulinaemic response study

Aim

The aim of this study is to examine the glycaemic response (GR) and insulinaemic response (IR) to:

- 50 g available carbohydrate Glucose
- 50 g available carbohydrate Glucose + 10 g **NUTRALYS®S85 Plus** pea protein
- 50 g available carbohydrate Glucose + 20 g **NUTRALYS®S85 Plus** pea protein
- 50 g available carbohydrate Glucose + 10 g Whey protein concentrate
- 50 g available carbohydrate Glucose + 20 g Whey protein concentrate

Study design

A single-blind, randomised, repeat measure, crossover design trial will be used to study the GR and IR following consumption of five products: one reference product (50 g available carbohydrate Glucose) and four test products (50 g available carbohydrate Glucose with 10 g **NUTRALYS®S85 Plus** pea protein, 50 g available carbohydrate Glucose with 20 g **NUTRALYS®S85 Plus** pea protein, 50 g available carbohydrate Glucose with 10 g Whey protein concentrate and 50 g available carbohydrate Glucose with 20 g Whey protein concentrate).

Participants will act as their own controls. Participants will be randomly assigned to test the products using a pseudo-random number generator. The trial will be conducted by the Oxford Brookes Centre for Nutrition and Health (OxBCNH). Ethical approval for the trial will be obtained from the University Research Ethics Committee (UREC) at Oxford Brookes University. Participants will be given full details of the study protocol and the opportunity to ask questions. All participants will give written informed consent prior to participation.

Participants

A sample size of 30 participants will be recruited. Healthy male and female adult participants (aged 18-60 years) will be recruited for the study. Participants will be excluded from the study if they meet any of the following criteria:

- Aged < 18 or > 60 years
- Pregnant or lactating
- Body mass index (BMI) > 30kg/m2
- Fasting blood glucose value > 6.1 mmol/l
- Any known food allergy or intolerance
- Medical condition(s) or medication(s) known to affect glucose regulation or appetite and/or which influence digestion and absorption of nutrients
- Known history of diabetes mellitus or the use of antihyperglycaemic drugs or insulin to treat diabetes and related conditions
- Major medical or surgical event requiring hospitalization within the preceding 3 months
- Use of steroids, protease inhibitors or antipsychotics (all of which have major effects on glucose metabolism and body fat distribution)

In addition, participants will be excluded if they are unable to comply with experimental procedures or do not follow GR and IR testing safety guidelines.

Products
Five products will be administered with 250 ml water:

- 50 g available carbohydrate Glucose
- 50 g available carbohydrate Glucose + 10 g **NUTRALYS®S85 Plus** pea protein
- 50 g available carbohydrate Glucose + 20 g **NUTRALYS®S85 Plus** pea protein
- 50 g available carbohydrate Glucose + 10 g Whey protein concentrate
- 50 g available carbohydrate Glucose + 20 g Whey protein concentrate

Protocol
The study will be conducted by Good Clinical Practise (GCP) certified researchers at OxBCNH. The protocol used by OxBCNH is based on ISO standards for the determination of the glycaemic index (GI).[1]

On the day prior to a test, participants will be asked to restrict their intake of alcohol and caffeine-containing drinks and to restrict their participation in intense physical activity (for example, long periods at the gym, excessive swimming, running and aerobics). Participants will also be told not to eat or drink after 9.00 pm the night before a test, although water will be allowed in moderation.

The reference product and four test products will be administered to participants in a randomised, repeated measures design. In each participant, the products will be tested in random order on separate days, with a one-week gap between measurements. Participants will be studied in the morning, before 10 am after a 12-hour overnight fast. Participants will consume the products at a comfortable pace, within 15 minutes and will remain sedentary during each session and will not consume any additional food or fluid.

Anthropometric measurements
All anthropometric measures will be taken, using standard methods, in the morning, after an overnight fast (12 hours):

- Height and weight
- BMI will be calculated using the standard formula: weight (kg)/height (m)^2
- Body composition using a body composition analyser (Tanita MC-980 MA; Tanita UK Ltd)

Glycaemic response (GR)
The glycaemic response method used by OxBCNH at Oxford Brookes University is carried out in accordance with the ISO 26642:2010 guidelines.[1]

Blood samples will be taken at -5 min and 0 min before consumption of the reference product/test products and the baseline value taken as a mean of these two values. The product will be consumed immediately after this and within 15 min. Further blood samples will be taken at 15, 30, 45, 60, 90, 120, 150 and 180 min after starting to drink. The time between participants finishing drinking and the first postprandial blood sample will be recorded.

[1] Food Products – Determination of the glycaemic index (GI) and recommendation for food classification. ISO 26642: 2010(E)

Blood will be obtained by finger-prick using the Unistik®3 single-use lancing device (Owen Mumford). Capillary blood sampling is preferred for reliable GR testing. Prior to a finger-prick, participants are encouraged to warm their hand to increase blood flow. Fingers are not squeezed to extract blood from the fingertip as this may dilute with plasma. Blood glucose will be measured using the HemoCue Glucose 201 DM analyser (HemoCue® Ltd), which is calibrated daily using a control solution from the manufacturer.
The incremental area under the blood glucose response curve (iAUC) will be calculated geometrically by applying the trapezoid rule, ignoring the area beneath the baseline.

Insulinaemic response (IR)

Blood samples in the fasting state will be obtained five minutes apart (-5 and 0 min) for measuring baseline insulin levels. Following the consumption of the reference product/test products, further blood samples will be collected at 15, 30, 45, 60, 90, 120, 150 and 180 min after starting to drink.

At each test time point, 300 μL of capillary blood (from finger pricks) will be obtained. Finger pricks will be made using the Unistik 3 single-use lancing device (Owen Mumford, Woodstock, UK) and blood will be collected into chilled Microvette® capillary blood collection tubes treated with di Pottasium EDTA (CB 300 K2E; Sarstedt Ltd., Leicester, UK). The Microvette® tubes will then be centrifuged and 200 μL of the supernatant plasma obtained. Insulin concentrations in the plasma samples will be determined by electrochemiluminescence immunoassay using an automated analyzer (Cobas® E411; Roche diagnostics, Burgess Hill, UK). The Cobas® system is a reliable method of plasma insulin determination[2]. The unit of measurement is μU/ml.

Statistical analysis

Data will be analysed using the IBM Statistical Package for the Social Sciences (SPSS, version 25). Data will be presented as mean, standard deviation (SD) and standard error of the mean (SEM) values. The blood glucose and plasma insulin iAUC (at 60, 90, 120 and 180 min) will be calculated geometrically by applying the trapezoid rule, according to the ISO standards. Prior to statistical analysis, the normality of the data will be tested using the Shapiro-Wilks statistic. Parametric or non-parametric tests will be used as appropriate to analyse the following results:

- blood glucose/insulin concentrations
- peak blood glucose/insulin value and time of the blood glucose/insulin peak
- glucose/insulin iAUC (at 60, 90, 120 and 180 min)

Comparisons will be made:

i) Between 50 g available carbohydrate Glucose, 50 g available carbohydrate Glucose + 10 g **NUTRALYS®S85 Plus** pea protein, 50 g available carbohydrate Glucose + 20 g **NUTRALYS®S85 Plus** pea protein, 50 g available carbohydrate Glucose + 10 g Whey protein concentrate and 50 g available carbohydrate Glucose + 20 g Whey protein concentrate

[2] Siahanidou T, Margeli A, Kappis A, Papassotiriou I & Mandyla H. Circulating visfatin levels in healthy preterm infants are independently associated with high-density lipoprotein cholesterol levels and dietary long-chain polyunsaturated fatty acids. *Metabolism* 2010;60(3):389-93

ii) Between 50 g available carbohydrate Glucose, 50 g available carbohydrate Glucose + 10 g **NUTRALYS®S85 Plus** pea protein and 50 g available carbohydrate Glucose + 20 g **NUTRALYS®S85 Plus** pea protein

Statistical significance will be set at $P < 0.05$.

Outcomes

- The primary outcome will be the GR and IR, as measured by the incremental area under the curve, blood glucose/insulin concentrations, peak and the time at which peaks occur
